# In Vitro Anti-HIV-1 Activity of Fucoidans from Brown Algae

**DOI:** 10.3390/md22080355

**Published:** 2024-07-31

**Authors:** Marina N. Nosik, Natalya V. Krylova, Roza V. Usoltseva, Valerii V. Surits, Dmitry E. Kireev, Mikhail Yu. Shchelkanov, Oxana A. Svitich, Svetlana P. Ermakova

**Affiliations:** 1 I.I. Mechnikov Institute of Vaccines and Sera, 105064 Moscow, Russia; svitichoa@yandex.ru; 2 G.P. Somov Institute of Epidemiology and Microbiology, Rospotrebnadzor, 690087 Vladivostok, Russia; adorob@mail.ru; 3 G.B. Elyakov Pacific Institute of Bioorganic Chemistry, Far Eastern Branch, Russian Academy of Sciences, 690022 Vladivostok, Russia; usoltseva-r@yandex.ru (R.V.U.); suritsw@yandex.ru (V.V.S.); 4 Central Research Institute of Epidemiology, Rospotrebnadzor, 111123 Moscow, Russia; dmitkireev@yandex.ru

**Keywords:** brown algae, fucoidans, HIV-1, anti-HIV-1 activity

## Abstract

Due to the developing resistance and intolerance to antiretroviral drugs, there is an urgent demand for alternative agents that can suppress the viral load in people living with human immunodeficiency virus (HIV). Recently, there has been increased interest in agents of marine origin such as, in particular, fucoidans to suppress HIV replication. In the present study, the anti-HIV-1 activity of fucoidans from the brown algae *Alaria marginata*, *Alaria ochotensis*, *Laminaria longipes*, *Saccharina cichorioides*, *Saccharina gurianovae*, and *Tauya basicrassa* was studied in vitro. The studied compounds were found to be able to inhibit HIV-1 replication at different stages of the virus life cycle. Herewith, all fucoidans exhibited significant antiviral activity by affecting the early stages of the virus–cell interaction. The fucoidan from *Saccharina cichorioides* showed the highest virus-inhibitory activity by blocking the virus’ attachment to and entry into the host’s cell, with a selectivity index (SI) > 160.

## 1. Introduction

Human immunodeficiency virus (HIV) infection is one of the most dangerous and widespread infectious diseases that has claimed more than 40 million human lives [1]. The global spread of HIV, which has assumed the character of a global epidemic, has made the problem of HIV infection a major health problem worldwide. According to the World Health Organization (WHO), by the end of 2022, there were 39 million people with HIV globally [1]. With the advent of antiretroviral therapy (ARV therapy), HIV infection ceased to be fatal and moved into the category of controlled chronic diseases [2,3]. To date, more than 40 antiretroviral drugs, aimed at blocking certain stages of the virus life cycle, are available for the treatment of HIV infection [4]. These drugs include nucleoside reverse transcriptase inhibitors (NRTIs), which block reverse transcriptase, an enzyme necessary for HIV to produce its own copies; non-nucleoside reverse transcriptase inhibitors (NNRTIs); protease inhibitors (PIs), an enzyme also necessary for the virus to produce its own copies; fusion inhibitors (FIs), blocking the entry of HIV into CD4+ cells; integrase inhibitors (INSTIs), blocking the enzyme integrase necessary for producing viral copies; attachment inhibitors (AIs), which bind to the gp120 protein on the outer surface of HIV, preventing HIV from entering CD4 cells; post-attachment inhibitors (PAIs), which block CD4 receptors on the surface of certain immune cells necessary for HIV entry; capsid inhibitors (CIs) acting on the HIV capsid; and CCR5 antagonists (EIs) blocking the CCR5 co-receptor on the surface of immune cells, which is necessary for the virus to enter cells [5,6,7,8,9]. In addition to the above-mentioned drugs, pharmacokinetic enhancers are also used to increase effectiveness of antiretroviral drugs included in the treatment regimen for HIV infection [3,5]. New drugs against HIV with fewer side effects, longer duration of action, and a reduced risk of drug resistance are rapidly being developed and introduced. However, despite such a wide range of antiretroviral drugs, there are individuals for whom HIV infection cannot be successfully treated with available medications due to the high degree of HIV-1 genetic variability [10,11] and its rapidly developing resistance and intolerance to drugs [12,13]. Moreover, in some people, drug resistance is detected even before the start of therapy. Such resistance can be either transmitted during infection or acquired during previous treatment. Thus, in women receiving antiretroviral treatment to prevent mother-to-child transmission of HIV, the virus’ resistance to a range of antiretroviral drugs of various classes is detected in 34–56% of cases [14,15]. Therefore, there is an urgent need to find alternative agents that can suppress viral load in people living with HIV.

Recently, there has been increased interest in agents of marine origin, such as, in particular, fucoidans, sulfated polysaccharides containing fucose (FCSP), to suppress HIV replication [16,17,18]. These polysaccharides consist mainly of repeating fucose units, but may also contain other sugars such as glucose, mannose, galactose, and uronic acid [18,19,20,21,22]. It has been discovered that many seaweed species contain significant amounts of complex structural sulfated polysaccharides (SPs) that have an inhibitory replicative activity against enveloped viruses [18]. In particular, recent studies have shown that brown algae of the orders *Fucales* and *Laminariales* are a vital source of fucoidans [16,17,23,24]. Sulfated polysaccharides containing fucose (FCSP) derived from these algae have anti-inflammatory, immunomodulatory properties, and HIV-inhibitory activity with various mechanisms of action [16,17,23,24]. The biological properties of fucoidans are closely related to their structure. Thus, their biological activity can be influenced by the content of fucose, the degree of sulfation, including replacement of monomeric links in the main chain, and also by the extraction procedure itself [16,17,23]. The activity of fucoidans is assumed to depend on both ionic changes and sugar rings, which spatially orient charges in a certain configuration and recognize the enzyme, thereby determining the specificity of binding [16,25]. The lack of a standardized procedure for obtaining fucoidans, the complex structure of fucoidans and, as a result, the differences in their biological characteristics hamper the development of medications based on them. It should be borne in mind that, to date, information on the biological properties of fucoidans derived from various marine sources is still quite limited.

Previously, our group showed that native (FeF) and enzyme-modified (FeHMP) fucoidans from the brown algae *Fucus evanescens* have HIV-inhibiting activity [26]. This makes further study of the antiviral effect of sulfated polysaccharides from other brown algae species to identify fucoidans with even stronger anti-HIV activity very promising. In the present study, we assessed the HIV-inhibitory activity of galactofucans, fucogalactans, and fucans derived from the following brown algae from the Sea of Okhotsk and Sea of Japan: *Alaria marginata*, *Alaria ochotensis*, *Laminaria longipes*, *Saccharina cichorioides*, *Saccharina gurianovae*, and *Tauya basicrassa*.

## 2. Results

The structural characteristics of the studied fucoidans from the brown algae *A. marginata*, *A. ochotensis*, *L. longipes*, *S. cichorioides*, *S. gurianovae*, and *T. basicrassa* are presented in Table 1.

A study of cytotoxicity of the investigated fucoidans by MTT assay showed that all polysaccharides (1–6) had low toxicity towards MT-4 cells: their 50% cytotoxic concentrations (CC_50_) exceeded 1000 µg/mL, while the CC_50_ of commercial drugs was within a range of 50–70 µg/mL (Table 2). Further analysis of the antiviral activity of the studied substances was carried out at concentrations below their CC_50_.

The antiviral effect of the compounds at different stages of the HIV-1 life cycle was assessed by analyzing inhibition of the cytopathogenic effect (CPE) of the virus. In addition to the destructive effect on cells, active replication of HIV-1 is characterized by the formation of large, multinucleated cells (syncytia) with their subsequent death. Therefore, the antiviral effect of the compounds was also assessed by analyzing the inhibition of syncytia formation in infected cells. The results of the analysis were used to calculate the 50% inhibitory concentration (IC_50_) and the selectivity index (SI = CC_50_/IC_50_) which indicates the effectiveness and safety of the compound (Table 2).

**Figure 1:**
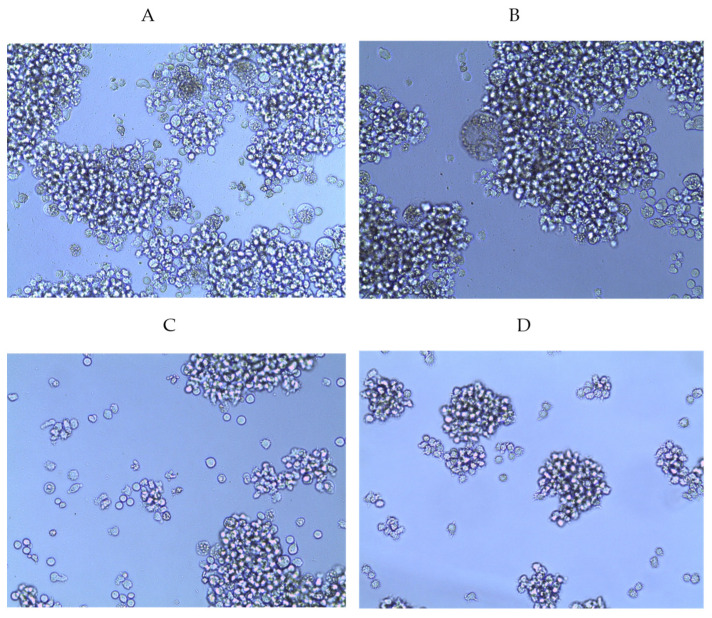

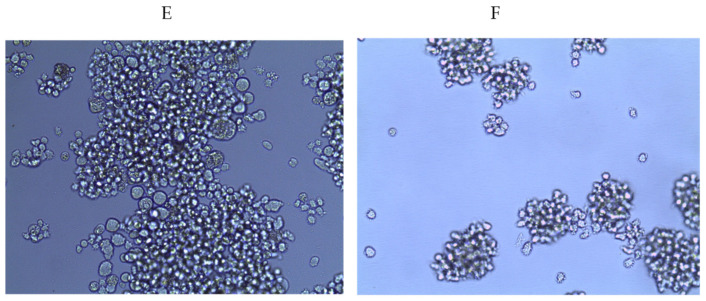
Dose-dependent antiviral activity of fucoidan **3** (LIF) on syncytia formation in HIV-infected MT-4 cells (pre-treatment of virus). (**A**) Concentration 25 µg/mL; (**B**) concentration 50 µg/mL; (**C**) concentration 100 µg/mL; (**D**) concentration 150 µg/mL; (**E**) virus control; (**F**) cell control.

We found that the studied fucoidans had antiviral activity, which was confirmed by the inhibition of the cytotoxic effect of the virus and the inhibition of syncytia formation (Table 2 and Table 3, Figure 1). A comparative study of the antiviral effect of some commercial drugs showed the highest antiviral activity for the PI drug (with the average values of the indices of 2.7 µg/mL (IC_50_) and approximately 26 (SI)), to which the anti-HIV activity of fucoidans was then compared (Table 2).

**Figure 2:**
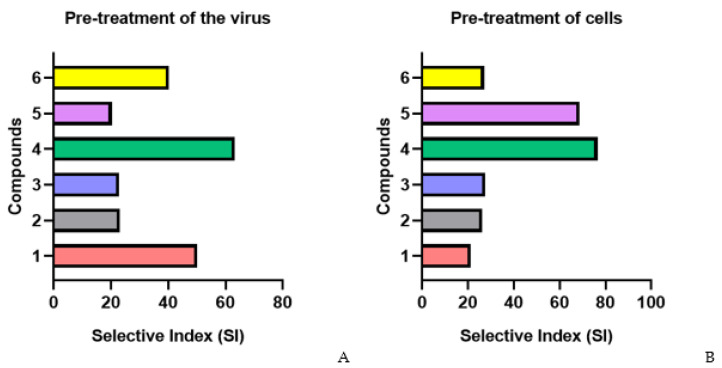

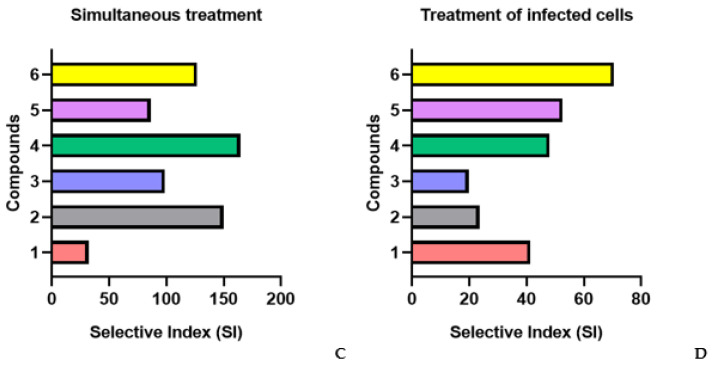
Anti-HIV-1 activity of studied fucoidans in various schemes of treatment with compound. (**A**) Pre-treatment of the virus; (**B**) pre-treatment of cells; (**C**) simultaneous treatment; (**D**) treatment of infected cells; **1**—AmF3; **2**—AoF3; **3**—LIF; **4**—ScF; **5**—SgF2; **6**—1TbF1.

The pretreatment of HIV-1 with fucoidans (direct virucidal effect) showed that several of the studied polysaccharides effectively inhibited viral replication (Figure 2A and Figure S1). Thus, the virucidal effect of compounds **1** (SI = 50.1), **4** (SI = 63.3), and **6** (SI = 40.3) was on average 2-fold higher than that of the reference PI drug (SI = 25.5) (*p* ≤ 0.05).

The pretreatment of cells with fucoidans (**1**, **2**, **3**, and **6**) before infection (preventive effect) showed their moderate antiviral activity (SI = 25.5) (Figure 2B). Compounds **4** (SI = 76.5) and **5** (SI = 68.7) more effectively (3.1–2.8-fold) protected cells from infection compared to the PI drug (SI = 24.6) (*p* ≤ 0.05).

The highest antiviral activity of fucoidans was observed at an early stage of infection (simultaneous exposure of cells to virus and compound) (Figure 2C). The selective index (SI) of all studied fucoidans (except for one of the compounds) exceeded that of the PI drug, on average, 4-fold (*p* ≤ 0.05). Compound **4** suppressed virus replication most effectively (5.3-fold) compared to PI (SI = 164.4 and SI = 31, respectively) (*p* ≤ 0.05).

In the case of treatment with fucoidans (**1**, **4**, and **6**) after adsorption and entry of the virus into cells (treatment of infected cells), moderate viral inhibitory activity of the compounds (SI = 53.3) was found, though it was 2.2-fold higher than that of the PI drug (SI = 24.6) (*p* ≤ 0.05) (Figure 2D).

## 3. Discussion

In this study, we assessed the HIV-1 antiviral activity of fucoidans derived from different species of brown algae from the Sea of Okhotsk and the Sea of Japan. The fucoidans differed primarily in the content of fucose and also in the degree of sulfation and monosaccharide composition. To assess the anti-HIV-1 activity of the obtained compounds, we used a replication-competent HIV-1 isolate. This is extremely important, since, unlike functional HIV-1 env clones (pseudoviruses) that have low replicative activity and cause minimal cytopathic effect in vitro, competently replicative viruses represent the complete HIV-1 genome. Accordingly, in an in vitro study with the use of these isolates, a completely replicative cycle is reproduced, identical to the replication cycle of the virus in vivo.

It is known that the antiviral activity of sulfated polysaccharides can affect various stages of the virus life cycle, including the inhibition of virus binding to cellular receptors, the prevention of virus penetration into cells, and the activation of intracellular signaling pathways [33,34]. Owing to the negatively charged sulfate groups, some of sulfated polysaccharides can suppress the infectious activity of the enveloped viruses by directly interacting with the viral particles [34,35]. The high SI of compounds **1** (AmF3), **4** (ScF), and **6** (1TbF1) during the pre-treatment of the virus indicate that in this case we may assume the inhibition of viral replication as a consequence of the direct effect of the studied compounds on the viral particles.

The detected significant decrease in viral activity with the simultaneous administration of five drugs (AoF3, LIF, ScF, SgF2, and 1TbF1) and infection of cells with HIV-1, indicates that the studied fractions of fucoidans block the fusion of the virus with the cell at an early stage of HIV-1 replication. The early contact of the virus with the target cell and the attachment of the virus to the cell occur due to the ionic interaction between positively charged external glycoproteins on the surface of the encapsulated virus and negatively charged components of the cell surface [33]. It has been shown that the interaction of negatively charged sulfate groups in fucoidans with positively charged amino acids of the HIV-1 envelope glycoprotein gp120 (in the V3 loop domain) prevents the virus from entering the cell [34,36]. Due to the structure of sulfated polysaccharides that is similar to the negatively charged glycosaminoglycan on the cell surface, fucoidans prevent the interaction of the virus with the target cell by competitive inhibition, forming a non-infectious fucoidan–virus complex, thereby preventing virus replication [33,34,36,37]. Our data on the antiviral activity of fucoidans against HIV-1, when they are administrated simultaneously with the infection of cells, are consistent with the results of other researchers who also showed that sulfated polysaccharides prevent HIV from entering target cells already at the early stages of the virus life cycle [18,19,38]. The suppression of syncytia formation also indicates that the studied compounds inhibit not only the adsorption of the virus but also the transmission of the virus from cell to cell. The inhibitory activity of sulfated polysaccharides isolated from various marine sources against the HIV-1-induced syncytia formation is confirmed by a number of other studies [39,40,41].

In the case of pretreatment of cells, two drugs (ScF and SgF2) showed high antiviral activity. Presumably, this may be due to the cellular receptors. The chemokine co-receptor CXCR4, along with other co-receptors, plays an extremely important role in the entry of HIV-1 into target cells [42,43,44]. Its expression correlates with increased viral replication and, as a result, rapid disease progression [43,44,45]. The identified ligand for CXCR4 is CXCL12 [46]. The binding of CXCL12 to CXCR4, along with leukocyte transport and homeostasis, activates various cellular functions [47,48,49]. It was previously shown that fucose-containing sulfated polysaccharides (FCSP) derived from brown algae can affect the CXCL12/CXCR4 axis by blocking CXCL12-induced activation of the CXCR4 receptor [50]. It was also demonstrated in vitro that fucoidans can have a negative effect on the percentage of CD34+ cells expressing the CXCR4 co-receptor [51]. In view of this finding, it can be assumed that the inhibition of viral activity in this case may be explained as follows: fucoidans downregulate the expression of the CXCR4 co-receptor on the surface of T cells, thereby making it difficult for the virus to attach to the cell and preventing its entry into the cell.

With treatment of infected cells, two compounds showed high inhibitory activity: **5** (SgF2) and **6** (1TbF1). Most likely, we here observe the suppression of the mechanisms of virus internalization and transcription and replication of the virus by the fucoidans. The virus internalization is a multi-stage process that involves the transport of the virus from the plasma membrane into the cell as a result of endocytosis and vesicular transport of the virus, followed by the release of viral RNA and its translocation into endosomes [52]. There is evidence that sulfated polysaccharides interfere with the internalization of the virus by interacting with proteins of the viral membrane [53]. In addition, sulfated polysaccharides, by attaching to the allosteric site of the viral capsid, can prevent the virus from entering the host cell [53]. The suppression of viral transcription and replication involves the direct effect of fucoidans on viral replication enzymes. Thus, it has been shown that polysaccharides derived from brown algae can inhibit reverse transcriptase [18]. It is reasonable to assume that fucoidans from other brown algae can also inhibit protease (an enzyme which, along with reverse transcriptase, plays a key role in HIV replication). Thus, with the treatment of HIV-1-infected cells, compounds **5** and **6** had an inhibitory effect simultaneously at the several stages of viral replication.

The highest antiviral activity in different administration schemes was shown by compound **4** (ScF), which was fucan with 100% fucose content. Fucans demonstrated a greater activity compared to similar polymers that consisted of galactose and glucose residues [38]. This was suggested to be due to the higher hydrophobic properties of fucose. However, compound **3** (LIF) that also contained 100% fucose showed moderate virus-inhibiting activity, which might be explained by the different types of linkages between fucose residues in ScF and LIF fucoidan molecules and, possibly, by the presence of unsulfated fucose residues in fucoidan LIF.

The observed differences in the antiviral activity of galactofucans, compounds **1** (AmF3) and **5** (SgF2) that are similar in type of main chain, structure and monosaccharide composition but differ in the degree of sulfation, once again confirm that the degree of sulfation plays an important role in manifestation of the biological properties of the fucoidans [16,19,36,54].

## 4. Materials and Methods

### 4.1. Cells

The cell line MT-4 (from the cell culture collection of the I.I. Mechnikov Institute of Vaccines and Sera, Moscow, Russia) were cultured in RPMI-1640 medium supplemented with 10% FBS (Sigma-Aldrich, Darmstadt, Germany, F9665), 2 mM glutamine, 100 U/mL of penicillin, and 100 U/mL of streptomycin.

### 4.2. Virus

In the study, we used the human immunodeficiency virus-1 (HIV-1) sub-subtype A6 (GenBank: BankIt2701146 VSMO71 OQ979188) from the collection of strains of human immunodeficiency viruses of the I.I. Mechnikov Institute of Vaccines and Sera, Moscow, Russia.

### 4.3. Fucoidan Extraction

We isolated polysaccharides from the brown algae *Alaria marginata* (Aa) (collected from the Sea of Okhotsk, Russia, in 2011), *Alaria ochotensis* (Ao) (collected from the Sea of Okhotsk, Russia, in 2003), *Laminaria longipes* (Ll) (collected from the Sea of Japan, Russia, in 1996), *Saccharina cichorioides* (Sc) (collected from the Sea of Okhotsk, Russia, in 2017), *Saccharina gurianovae* (Sg) (collected from the Sea of Okhotsk, Russia, in 2003), and *Tauya basicrassa* (Tb) (collected from the Sea of Okhotsk, Russia, in 2014) by the methods for integrated processing of algae and production of water-soluble polysaccharides, developed in the G.B. Elyakov Institute of Bioorganic Chemistry, Far Eastern Branch, Russian Academy of Sciences [55], with some modifications.

Briefly, a sample of defatted, dried, and powdered algal frond (*A. marginata* (Aa), *A. ochotensis* (Ao), *L. longipes* (Ll), *S. cichorioides* (Sc), *S. gurianovae* (Sg), and *T. basicrassa* (Tb)) were treated with 70% ethanol to separate low-molecular-weight substances. Then the algae were dried and the polysaccharides were extracted with a solution of hydrochloric acid (60 °C). The extracts were collected, combined, concentrated on a rotary evaporator, dialyzed against distilled water, and freeze-dried. Polysaccharides (P) were isolated from dried extracts by ion exchange chromatography on a DEAE Macro-Prep column. The samples (1 g) of polysaccharides (AoP, LlP, ScP, SgP, and TbP) were dissolved in 0.04 N HCl (1 g/20 mL), centrifugated, and the supernatant was applied to a DEAE Macro-Prep column (13 × 4 cm). Neutral polysaccharides were eluted from the column with 0.04 M NaCl (until the eluate reacted negatively to carbohydrate content). Then, the column was eluted with a linear gradient of NaCl (from 0.1 M to 2 M) using EconoPump and a Model 2110 Fraction Collector (Bio Rad Laboratories, Inc., Hercules, CA, USA). Analysis of fractions for carbohydrate content was carried out using the phenol-sulfuric acid method [56].

The resulting fractions were concentrated on a Buchi Rotavapor R-114 rotary evaporator (Switzerland) (up to ~100 mL), dialyzed against water for 24 h, and freeze-dried. As a result, fucoidan fractions AmF3, AoF3, LlF, ScF, SgF2, 1TbF1 were obtained from *A. marginata*, *A. ochotensis*, *L. longipes*, *S. cichorioides*, *S. gurianovae* and *T. basicrassa* with yields of 0.4, 2.2, 0.35, 2.2, 2.0, and 0.21%, respectively, of the dry weight of defatted seaweed.

The number of sulfate groups was determined by the BaCl_2_ gelatin method [57].

Acid hydrolysis of polysaccharides. For hydrolysis, 5 mg of the polysaccharide fraction was dissolved in 500 μL of 2N TFA. Hydrolysis was carried out at 100 °C for 8 h. After the hydrolysis, the acid was distilled off on a rotary evaporator three times with ammonia.

The monosaccharide composition of polysaccharides was determined after acid hydrolysis using a Shim-pack ISA-07/S2504 column (0.4 × 25 cm). Then the sample was eluted with potassium borate buffer at an elution rate of 0.6 mL/min. Detection of monosaccharides was carried out by the bicinchoninate method on a Shimadzu C-R2 AX integrating system. Monosaccharides (Man, Rha, Fuc, Gal, Xyl, and Glc) were used as standards [58].

### 4.4. Compounds

#### 4.4.1. Studied Compounds (Fucoidans)

(**1**) AmF3 from *Alaria marginata* is a sulfated and acetylated branched galactofucan with a main chain of →3)-α-L-Fucp-(2,4SO^3−^)-(1→ repeating units. Chains of galactose residues (DP up to 9) were found in AmF3 fucoidan [27];

(**2**) AoF3 from *Alaria ochotensis* is a complex branched fucogalactan containing mainly 1,3- and 1,4-linked galactose residues and 1,3-linked fucose residues [28];

(**3**) LlF from *Laminaria longipes* is a sulfated fucan containing predominantly →3)-α-L-Fucp-(2SO^3–^)-(1→4)-α-L-Fucp-(1→2)-α-L-Fucp-(4SO^3–^)-(1→ repeating units, with small amounts of disaccharide 1,4-linked fragments and 3-sulfated fucose residues [29];

(**4**) ScF from *Saccharina cichorioides* is a sulfated fucan with a main chain of 2,4-sulfated 1,3-linked α-L-fucopyranose residues, a small amount of 1,4-linked α-L-fucopyranose residues, and branches at C2 in the form of single α-L-fucose residues [30];

(**5**) SgF2 from *Saccharina gurianovae* is a sulfated and acetylated galactofucan containing a backbone from →3)-α-L-Fucp-(2,4SO^3−^)-(1→ repeating units. Shorter (1→4)- and/or (1→6)-linked sulfated galactose chains are attached at positions C2, C3 of fucose residues [31];

(**6**) 1TbF1 from *Tauya basicrassa* is a sulfated and acetylated fucogalactan containing a backbone from 1,6-linked residues of β-D-galactopyranose with branches at C3 and C4, terminal fucose and galactose residues and fragments from 1,3-; 1,4-; and 1,2-fucose residues [32].

#### 4.4.2. Reference (Commercial) Antiretroviral Drugs

(1) Non-nucleoside reverse transcriptase inhibitor (NNRTI): Etravirine (ETR), (Product # SML2597, Merck Life Science, LLC, Darmstadt, Germany); (2) Nucleoside reverse transcriptase inhibitor (NRTI): Stavudine (d4T), (Product # Y0001683, Merck Life Science, LLC, Darmstadt, Germany); (3) Integrase strand transfer inhibitor (INSTI): Raltegravir (RAL), (Product # SML3670, Merck Life Science, LLC, Darmstadt, Germany); (4) Protease inhibitor (PI): Indinavir (IDV), (Product # SML0189, Merck Life Science, LLC., Darmstadt, Germany).

### 4.5. Cytotoxic Activity of Fucoidans

The cytotoxicity assay of the studied compounds was carried out on the MT-4 lymphoblastoid cell line and evaluated using the MTT assay, as described previously [52]. In brief, the cells were incubated for 144 h at 37 °C in an atmosphere with 5% CO_2_ and 98% humidity in 96-well plates with drugs at various concentrations (25–350 μg/mL). Untreated MT-4 cells served as a control. Then, the MTT solution was removed, and isopropanol was added to dissolve the insoluble formazan crystals. Optical density (OD) of the dissolved formazan was measured at 540 nm using an ELISA microplate reader (Labsystems Multiskan RC, Vantaa, Finland) with a reference absorbance at 620 nm. Cell viability was calculated as (ODo)/(ODc) × 100%, where ODo and ODc are the optical densities of the treated and control cells, respectively. The 50% cytotoxic concentration of the drug (CC_50_), which reduces the viability of the treated cells by 50% compared to the control, was calculated using regression analysis of dose-dependent curves [59].

### 4.6. Antiviral Activity of Fucoidans

The antiviral activity of the compounds against HIV-1 was determined by the methods for inhibition of the cytopathogenic effect (CPE) of the virus and inhibition of the syncytia formation in MT-4 cells. Suspension of MT-4 cells was cultured in 96-well plates at a concentration of 2 × 10^5^ cells per well with the following infection by HIV-1 (0.001 TCID_50_/cell). Several schemes for the use of fucoidans and reference drugs were tested, with each scheme performed in triplicate using triplets of different concentrations of compounds (25–350 µg/mL). The plates were incubated at 37 °C in a CO_2_ incubator for 144 h up to 80–90% CPE in virus control compared to cell control. Syncytia formation was monitored starting from day 2 post-infection.

Pretreatment of virus with fucoidans. The virus was added to various concentrations of compounds at a ratio of 1:1 (*v*/*v*), incubated for 1 h at 37 °C, and then added to the cells. After 1 h of adsorption at 37 °C, the cells were washed twice with 1× phosphate-buffered saline (PBS) and incubated in the maintenance medium until the appearance of CPE.

Cell pretreatment with fucoidans. The cells were treated with various concentrations of compounds for 2 h at 37 °C. Then the cells were washed twice with 1× PBS and infected with the virus, following incubation for 1 h at 37 °C. Afterwards the cells were washed twice with 1× PBS to remove the unsorbed virus and incubated in the maintenance medium until the appearance of CPE.

Simultaneous treatment of cells with fucoidans and virus. The cells were infected with the virus and simultaneously treated with various concentrations of compounds (at a ratio of 1:1) for 1 h at 37 °C. The cells were then washed twice with 1× PBS and incubated in the maintenance medium until the appearance of CPE.

Treatment of infected cells with fucoidans. The cells were infected with the virus, incubated for 1 h at 37 °C, then the cells were washed twice with 1× PBS, treated with various concentrations of fucoidans, and incubated in the maintenance medium until the appearance of CPE.

To evaluate the antiviral activity of fucoidans, we used the MTT assay as described above. After the incubation, the antiviral activity of the polysaccharides and the reference drugs was evaluated by the rate of inhibition (IR, %) of the cytopathogenic effect (CPE) of HIV-1 in the cell culture. IR was calculated using the formula:

IR = (ODtv − ODcv)/(ODcd − ODcv) × 100%,

where ODtv is the optical density (OD) of infected cells treated with compounds; ODcv is the OD of control infected cells; ODcd is the OD of control (uninfected) cells. The inhibitory concentration (IC_50_) was defined as the concentration of a compound that reduced the virus-induced CPE by 50% [59]. The selectivity index (SI) of the compound was calculated as the ratio of CC_50_ to IC_50_.

### 4.7. Statistical Analysis

Data were analyzed using the Statistica 10.0 software package (StatSoft, Inc., Tulsa, OK, USA). The CC_50_ and IC_50_ values were calculated using the linear regression analysis of the dose-effect curve. The results are presented as the mean and standard deviation in three or more independent experiments. The differences between the indicators of the control and experimental groups were compared using the Wilcoxon test for related samples. Differences were considered significant at a level of *p* ≤ 0.05.

## 5. Conclusions

The data we obtained show that the studied fucoidans (compounds **1**–**6**) from the brown algae *Alaria marginata*, *A. ochotensis*, *Laminaria longipes*, *Saccharina cichorioides*, *S. gurianovae*, and *Tauya basicrassa* are able to inhibit the HIV-1 replication at different stages of the virus life cycle. At the same time, they have low cytotoxicity, which leads to high selectivity. Since the tested compounds exhibited the highest antiviral activity (except for compound **3** (LIF)) when the cells were simultaneously exposed to fucoidans and the virus, we may assume that the mechanisms underlying their antiviral effect are primarily associated with blocking of the virus’ attachment to and entry into the host cell. Fucoidan **4** (ScF) with 100% fucose content turned out to be the most effective polysaccharide because it showed high antiviral activity under different schemes of compounds administration. 

## Figures and Tables

**Table 1:** Yields and structural characteristics of fucoidans from the brown algae *A. marginata* (AmF3), *A. ochotensis* (AoF3), *L. longipes* (LlF), *S. cichorioides* (ScF), *S. gurianovae* (SgF2), and *T. basicrassa* (1TbF1).

Fucoidan	Yield, % *	SO_3_Na, % **	Monosaccharide Composition, mol %				Reference
			Fuc	Gal	Man	Xyl	
AmF3	0.4	28	47.5	47.3	0	5.2	[27]
AoF3	2.2	34	22	78	0	0	[28]
LlF	0.35	32	100	0	0	0	[29]
ScF	2.2	39	100	0	0	0	[30]
SgF2	2.0	38	50	40.6	2.0	0	[31]
1TbF1	0.21	21	42.4	57.6	0	0	[32]

* % of dry defatted algae; ** % of sample weight.

**Table 2:** Anti-HIV activity of different types of fucoidans and reference drugs.

Compound	CC_50_ (µg/mL)	Pre-Treatment of Virus		Pretreatment of Cells		Simultaneous Treatment		Treatment of Infected Cells	
		IC_50_ (µg/mL)	SI	IC_50_ (µg/mL)	SI	IC_50_ (µg/mL)	SI	IC_50_ (µg/mL)	SI
**1** (AmF3)	1058 ± 116	21.1 ± 2.5	50.1 ± 6.5 *	49.8 ± 6.5	21.2 ± 2.7	33.1 ± 4.3	32.0 ± 4.2	25.6 ± 3.3	41.3 ± 5.4 *
**2** (AoF3)	2395 ± 263	103.5 ± 12.4	23.1 ± 2.8	91.3 ± 11.0	26.2 ± 3.1	16.0 ± 2.1	149.7 ± 16.5 *	101.9 ± 11.2	23.5 ± 3.3
**3** (LlF)	1509 ± 181	66.2 ± 8.6	22.8 ± 3.0	54.6 ± 7.1	27.6 ± 3.6	15.3 ± 2.0	98.6 ± 10.8 *	76.3 ± 9.1	19.8 ± 2.6
**4** (ScF)	2203 ± 242	34.8 ± 4.5	63.3 ± 8.2 *	28.8 ± 3.4	76.5 ± 9.2 *	13.4 ± 1.7	164.4 ± 18.1 *	45.9 ± 6.0	48.0 ± 6.2 *
**5** (SgF2)	2232 ± 245	110.0 ± 13.0	20.3 ± 2.4	32.5 ± 3.9	68.7 ± 8.2 *	25.9 ± 3.4	86.2 ± 11.2 *	42.4 ± 5.5	52.6 ± 6.8 *
**6** (1TbF1)	2523 ± 278	62.6 ± 7.5	40.3 ± 5.2 *	93.0 ± 10.2	27.1 ± 3.5	19.9 ± 2.6	126.8 *	35.8 ± 5.0	70.5 ± 9.2 *
NNRTI	54 ± 7	7.9 ± 1.0	6.8 ± 0.9	9.0 ± 1.2	5.9 ± 0.7	7.9 ± 1.0	6.7 ± 0.8	2.9 ± 0.4	18.6 ± 2.4
NRTI	51 ± 7	11.6 ± 1.4	4.4 ± 0.5	10.2 ± 1.3	4.9 ± 0.6	5.4 ± 0.7	9.4 ± 1.0	2.2 ± 0.3	23.0 ± 3.2
INSTI	63 ± 8	3.1 ± 0.4	20.2 ± 2.6	4.4 ± 0.5	14.2 ± 1.7	1.9 ± 0.2	32.9 ± 3.9	2.0 ± 0.3	31.3 ± 4.4
PI	71 ± 9	2.8 ± 0.3	25.5 ± 3.3	2.9 ± 0.3	24.6 ± 2.9	2.3 ± 0.3	31.0 ± 4.0	2.9 ± 0.4	24.6 ± 3.2

CC_50_—50% cytotoxic concentration of compounds; IC_50_—50% virus inhibitory concentration of compounds; SI—selective index of compounds (CC_50_/IC_50_); *—statistically significant difference between PI (a commercial drug with the highest SI) and the tested fucoidans (*p* ≤ 0.05).

**Table 3:** Impact of different types of fucoidans on syncytia formation in HIV-infected MT-4 cells.

Fucoidan	Concentration, µg/mL	Pretreatment of Cells	Pretreatment of Virus	Simultaneous Treatment	Treatment of Infected Cells
**1** (AmF3)	25	+	-	-	+
	50	-	-	-	-
	100	-	-	-	-
	150	-	-	-	-
**2** (AoF3)	25	+	+ +	-	+ +
	50	-	+ +	-	+ +
	100	-	+ +	-	-
	150	-	-	-	-
**3** (LlF)	25	-	+ +	+	+ +
	50	-	+ +	-	+ +
	100	-	+	-	-
	150	-	-	-	-
**4** (ScF)	25	+	+ +	+	+ +
	50	-	+ +	-	+ +
	100	-	-	-	-
	150	-	-	-	-
**5** (SgF2)	25	+	+ +	+	+ +
	50	-	+ +	-	+ +
	100	-	++	-	-
	150	-	-	-	-
**6** (1TbF1)	25	+	-	-	+
	50	-	-	-	-
	100	-	-	-	-
	150	-	-	-	-

-: absence of syncytia; +: presence of single syncytia; ++: multiple syncytia. Since the inhibition of syncytia formation was observed starting from a concentration of 150 µg/mL, higher concentrations of the studied compounds (200, 250, 300 and 350 µg/mL) are not indicated in the table.

## Data Availability

The data presented in this study are available in this article and Supplementary Materials.

## Supplementary Materials

The following supporting information can be downloaded at: https://www.mdpi.com/article/10.3390/md22080355/s1, Figure S1: Anti-HIV-1 activity of different fucoidans (direct virucidal effect).
