# Supplementary material for: In Vitro Anti-HIV-1 Activity of Fucoidans from Brown Algae
Source: Mar Drugs. 2024 Jul 31;22(8):355. doi: 10.3390/md22080355 (PMC11355542; doi:10.3390/md22080355)
Supplement: Supplementary file 1 [file marinedrugs-22-00355-s001.zip › marinedrugs-3104892-supplementary.pdf]

Supplementary material

In Vitro Anti-HIV-1 Activity of Fucoidans from Brown Algae

Marina N. Nosik [1,*,†], Natalya V. Krylova [2,*,†], Roza V. Usoltseva [3], Valerii V. Surits [3], Dmitry E. Kireev [4], Mikhail Yu. Shchelkanov [2], Oxana A. Svitich [1] and Svetlana P. Ermakova [3,*]

[1] I.I. Mechnikov Institute of Vaccines and Sera, 105064 Moscow, Russia; svitichoa@yandex.ru
[2] G.P. Somov Institute of Epidemiology and Microbiology, Rospotrebnadzor, 690087 Vladivostok, Russia; adorob@mail.ru
[3] G.B. Elyakov Pacific Institute of Bioorganic Chemistry, Far Eastern Branch, Russian Academy of Sciences, 690022 Vladivostok, Russia; usoltseva-r@yandex.ru (R.V.U.); suritsw@yandex.ru (V.V.S.)
[4] Central Research Institute of Epidemiology, Rospotrebnadzor, 111123 Moscow, Russia; dmitkireev@yandex.ru
* Correspondence: mnossik@yandex.ru (M.N.N.); krylovanatalya@gmail.com (N.V.K.); swetlana_e@mail.ru (S.P.E.)
† These authors contributed equally to this work.

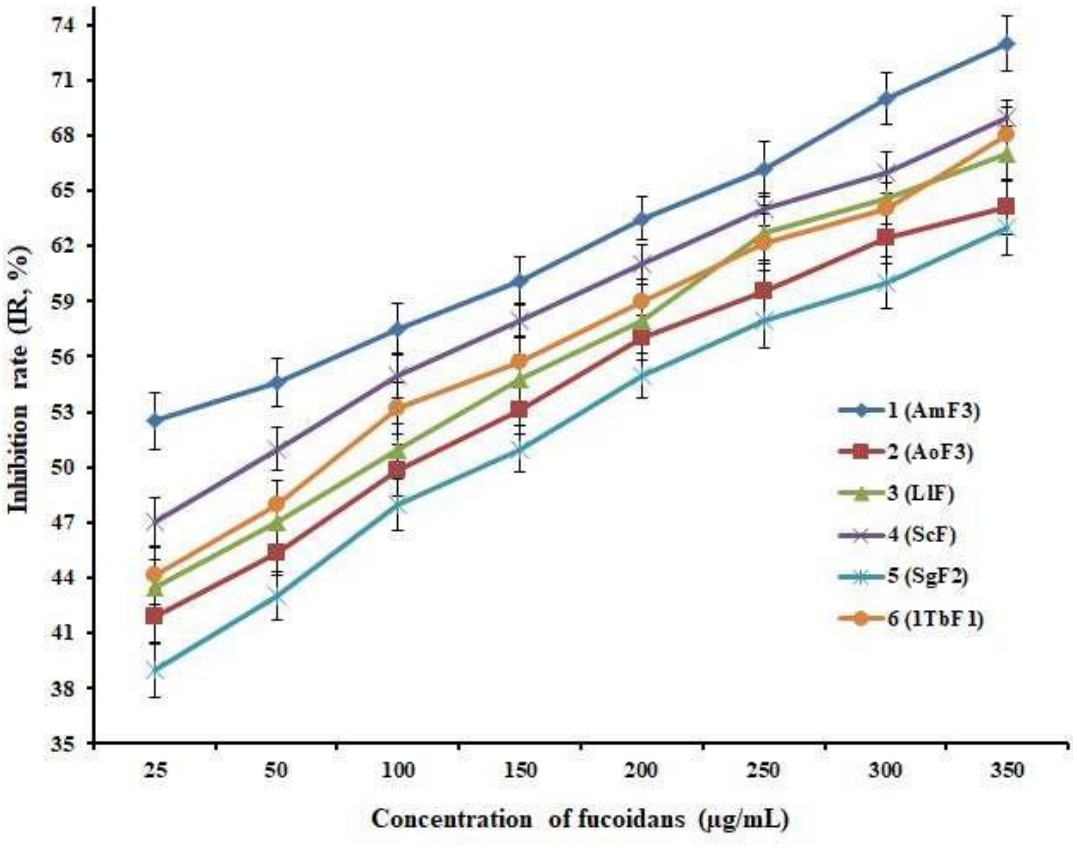

Figure S1. Anti-HIV-1 activity of different fucoidans (direct virucidal effect). The virus was added to various concentrations of compounds (25-350 μg/ml), incubated for 1 hour at 37 °C and then added to the MT-4 cells. Data are presented as the inhibition rate (IR, %) of the cytopathogenic effect (CPE) of the virus by fucoidans. The inhibitory concentration (IC50) values of the compounds were calculated by regression analysis of the dose–response curves. The results include data from three experiments (mean ± SD).
